# Enhancing yield prediction in maize breeding using UAV-derived RGB imagery: a novel classification-integrated regression approach

**DOI:** 10.3389/fpls.2025.1511871

**Published:** 2025-03-20

**Authors:** Haixiao Ge, Qi Zhang, Min Shen, Yang Qin, Lin Wang, Cansheng Yuan

**Affiliations:** ^1^ College of Rural Revitalization, Jiangsu Open University, Nanjing, China; ^2^ Institute of Agricultural Resources and Environment, Jiangsu Academy of Agricultural Sciences, Nanjing, China

**Keywords:** maize, yield prediction, UAV-based imagery, random forest, pre-regression classification

## Abstract

Accurate grain yield prediction is crucial for optimizing agricultural practices and ensuring food security. This study introduces a novel classification-integrated regression approach to improve maize yield prediction using UAV-derived RGB imagery. We compared three classifiers—Support Vector Machine (SVM), Decision Tree (DT), and Random Forest (RF)—to categorize yield data into low, medium, and high classes. Among these, SVM achieved the highest classification accuracy and was selected for classifying data prior to regression. Two methodologies were evaluated: Method 1 (direct RF regression on the full dataset) and Method 2 (SVM classification followed by class-specific RF regression). Multi-temporal vegetation indices (VIs) were analyzed across key growth stages, with the early vegetative phase yielding the lowest prediction errors. Method 2 significantly outperformed Method 1, reducing RMSE by 45.1% in calibration (0.28 t/ha vs. 0.51 t/ha) and 3.3% in validation (0.89 t/ha vs. 0.92 t/ha). This integrated framework demonstrates the advantage of combining classification and regression for precise yield estimation, providing a scalable tool for maize breeding programs. The results highlight the potential of UAV-based phenotyping to enhance agricultural productivity and support global food systems.

## 1 Introduction

Accurate crop yield prediction is essential for optimizing agricultural decisions, including harvest planning, crop insurance, and storage management. Reliable yield forecasts are crucial for farmers, agronomists, and agricultural policymakers to ensure efficient resource allocation and enhance productivity. Traditional yield estimation methods typically rely on field sampling, which is labor-intensive, destructive, and prone to inaccuracies (Liang et al., 2024). These methods usually involve collecting samples from the field and analyzing them to estimate the overall yield. However, this process is not only time-consuming but also disruptive to the growing crop. Additionally, the experiential knowledge of farmers and agricultural technicians is often used to predict crop yield, but this approach remains subjective and can be prone to errors, especially in large-scale or diverse farming systems (Zhang et al., 2020). As a result, yield estimates based on such knowledge can vary significantly, lacking consistency and often leading to inaccurate predictions.

To address these challenges, remote sensing technologies have emerged as powerful tools in modern agriculture. Over the past decade, the development of high-throughput phenotyping (HTP) systems—based on both ground-based mobile platforms and aerial systems—has revolutionized how we monitor crop growth and predict yield. These systems provide high spatial and temporal resolution data, which can be directly related to grain yield or crop responses to both biotic and abiotic stresses (Feng et al., 2020). Among these technologies, the application of unmanned aerial vehicles (UAVs) with high spatial resolution imagery has shown considerable success in estimating crop yield, particularly through the use of vegetation indices (VIs), such as the Normalized Difference Vegetation Index (NDVI) (Ballesteros et al., 2014; Candiago et al., 2015). These indices have been demonstrated to correlate strongly with crop yield, making them an effective tool for monitoring crop health and predicting yield (Hassan et al., 2019). Numerous statistical methods have been employed to estimate agricultural variables from UAV-derived VI data. Linear regression models are commonly used to calibrate the relationship between UAV-based VIs and measured agricultural variables, such as crop height and yield. For instance, Geipel et al. (2014) utilized UAV-RGB imagery to predict corn grain yield by calculating crop height and VIs. Similarly, Vega et al. (2015) found a strong linear relationship between NDVI and yield when extracting NDVI data from multi-temporal UAV imagery.

While these methods are useful, they also have limitations. One major drawback is their inability to account for the complex relationships between multiple variables involved in crop growth and yield. Traditional regression models may oversimplify these relationships, leading to lower accuracy in yield prediction (Duan et al., 2021). In contrast, machine learning techniques have gained popularity due to their ability to model complex, non-linear relationships between numerous variables without relying on explicit equations. For instance, machine learning models such as support vector machines (SVM) and random forests (RF) have been successfully applied to estimate crop yield by integrating various input features like weather conditions, soil properties, and remote sensing data (Cai et al., 2019). These models can handle large and high-dimensional datasets, making them well-suited for real-time yield prediction in precision agriculture. By capturing intricate interactions between environmental factors, crop physiology, and management practices, machine learning models are able to provide more robust predictions than traditional methods. Moreover, machine learning algorithms can be trained to adapt to new data, improving their accuracy over time as more information becomes available. This adaptability makes machine learning particularly valuable in agricultural settings, where conditions and inputs vary widely across regions and seasons.

Maize (*Zea mays* L.) stands as a global staple crop with triple significance in food security, bioenergy production, and livestock nutrition (Herrmann et al., 2020). In breeding programs, the annual yield testing protocol for new cultivars demands particularly accurate prediction methodologies, as even marginal improvements in estimation accuracy can substantially accelerate cultivar development cycles. Traditional machine learning regression approaches for yield prediction, however, often face limitations when dealing with the inherent heterogeneity across diverse maize cultivars and environmental interactions. Recent advances in two-stage analytical frameworks combining classification with regression have demonstrated remarkable success in spectral analysis applications. Notably, Wang et al. (2014) achieved enhanced coal property predictions through initial spectral classification, while Wang et al. (2019) improved glucose content estimation via categorical preprocessing of spectral data. These successes suggest that a classification-before-regression approach could effectively address the spectral complexity and cultivar variability challenges in maize yield prediction. By stratifying populations into homogeneous subgroups before applying subgroup-specific regression models, this methodology minimizes inter-group interference while maximizing intra-group pattern recognition - a critical advantage for precision agriculture applications.

This study introduces three significant advancements to maize yield prediction research. First, we establish a novel two-stage framework involving initial yield potential classification using UAV-derived RGB imagery followed by subgroup-optimized regression modeling. Second, we systematically investigate the temporal dynamics of VIs across critical growth stages and their cultivar-specific relationships with final yield - an aspect previously under characterized in maize phenomics. Third, through comprehensive comparison with conventional regression techniques, we demonstrate the superior performance of our stratified approach in handling cultivar diversity.

The manuscript is organized as follows: Section 1 provides a comprehensive review of the relevant literature on crop yield estimation, focusing on UAVs and machine learning techniques. Section 2 outlines the methodology used in this study, including data collection, feature extraction, and the application of classification and regression procedures. Section 3 presents the results and performance evaluation of the proposed framework. Section 4 discusses the implications of the findings, addresses the limitations of the current study, and offers recommendations for future research directions. Finally, Section 5 presents the conclusion, summarizing the key findings and emphasizing the potential impact of the proposed classification-before-regression technique on improving yield estimation accuracy in maize breeding programs.

## 2 Materials and methods

### 2.1 Experimental location and plant materials

**Figure 1:**
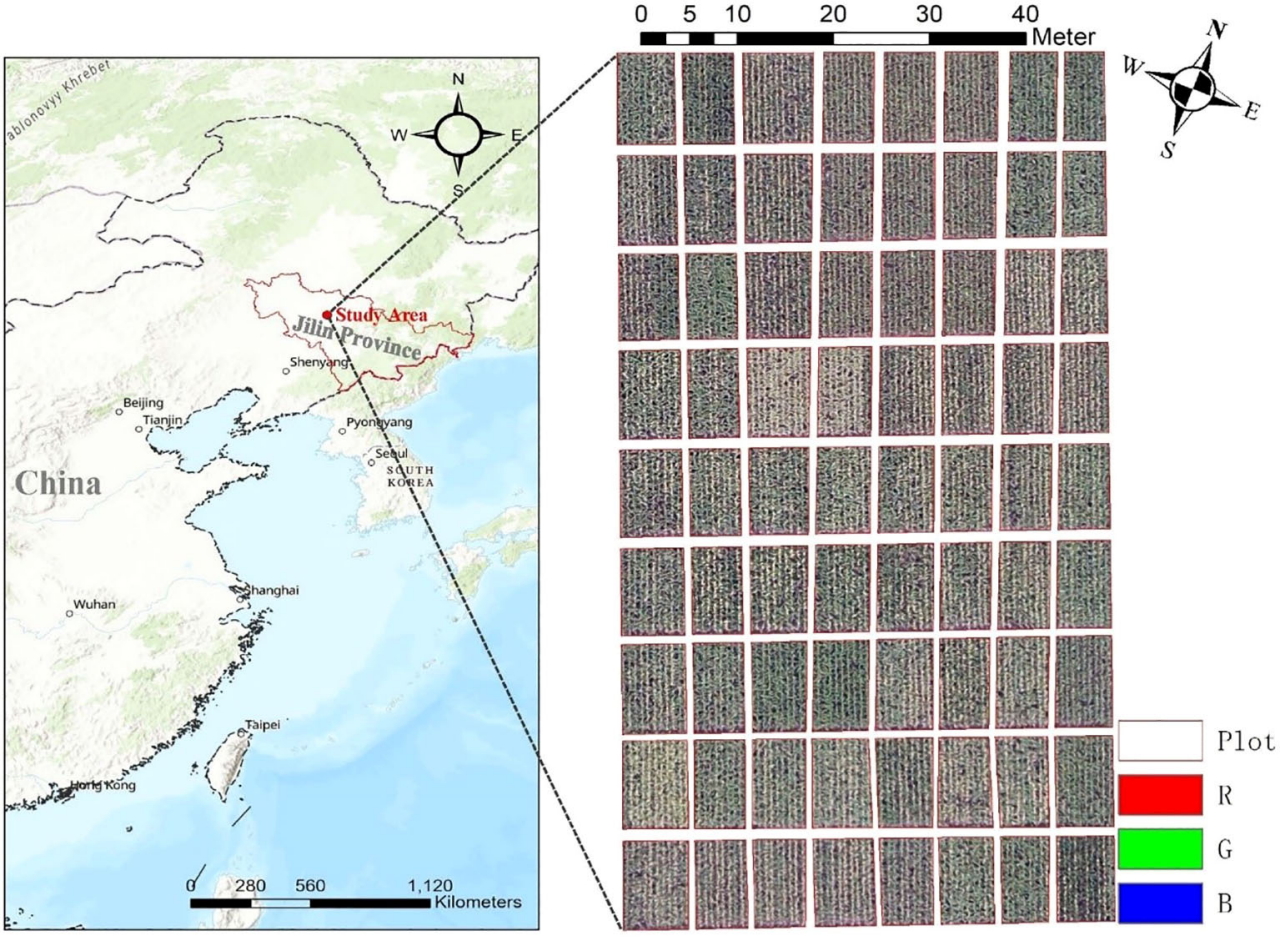
Location of the study area and overview of the experimental field.

This study was conducted at a maize breeding base in Nong’an County, Changchun City, Jilin Province, China (125°8’28’’ E, 44°22’25’’ N), situated within the fertile black soil (Chernozem) region of the Songnen Plain, a key maize-producing area in Northeast China (**Figure 1**). The experimental site featured flat terrain and uniform soil properties optimized for high-yield maize cultivation. A total of 72 plots (5.0 m × 6.0 m each) were arranged in a randomized complete block design, with eight rows per plot, 60 cm row spacing, and 25 cm plant spacing. Forty-two maize genotypes were selected from the core germplasms of the Jilin Academy of Agricultural Sciences (JAAS), representing elite inbred lines, widely adopted hybrids, and locally adapted landraces. These genotypes were sown on May 2, 2021 (**Table 1**), aligning with the optimal planting window for maize in Jilin Province. They were chosen for their adaptability to Jilin’s temperate climate—including tolerance to early-season cold stress and resistance to prevalent diseases (e.g., northern leaf blight, stalk rot)—genetic diversity, and alignment with regional breeding objectives such as yield stability, abiotic stress resilience, and dual-purpose (grain/silage) utility. The planting density was standardized at 75,000 plants per hectare, consistent with local high-yield practices. Field management followed regional protocols: a base application of 200 kg/ha compound fertilizer (15:15:15 NPK) at sowing, 150 kg/ha urea topdressing at the V6 stage, supplemental irrigation during critical growth phases, and integrated pest management to minimize biotic stressors.

**Table 1:** Details of the experiment and UAV flights in 2021.

Rice plots	Date of sowing	Date of maturity	Date of UAV flights
72	2 May	23 September-6 October	9 July, 21 July, 27 July, 5 August, 14 August, 25 August, 4 September, 18 September, 28 September

### 2.2 Data collection

#### 2.2.1 Yield collection

At the end of the maize growing season (late September to early October), all maize plots under investigation were harvested manually. In each plot, all the maize plants were harvested, and the total grain yield was measured. To minimize the effects of plot boundaries, the entire area of each 5.0 m × 6.0 m plot was included in the harvest, ensuring that data collected from the entire plot represented the full yield potential of the genotype being evaluated.

The harvested cobs were threshed, and the grains were dried to a moisture content of approximately 12%. The dried grains were then weighed using an electronic scale with an accuracy of ± 0.1 g. The final yield was converted to kilograms per hectare (kg/ha) based on the plot area. This method, where the entire plot is harvested, helps reduce the potential bias from edge effects and provides a more accurate reflection of the genotype’s performance across the entire plot. The grain yield was then used as the target variable for training and testing the yield prediction models.

#### 2.2.2 UAV image acquisition

The acquisition dates of UAV-based images are provided in **Table 1**. In this study, the UAV-based remote sensing system comprised a consumer-grade RGB camera and a UAV platform (Phantom 4 Pro, DJI, Shenzhen, China). Under optimal conditions, the UAV system could hover for up to 30 minutes. The RGB camera was equipped with a one-inch complementary metal-oxide-semiconductor (CMOS) sensor, capable of capturing still images with a spatial resolution of approximately 20 million pixels. To ensure image quality, the RGB camera was positioned vertically downward during each flight. The flight elevation was set to 50 m, resulting in a ground sampling distance (GSD) of 1.36 cm/pixel. The UAV control app (Pix4Dcapture, Pix4D Corporation, Lausanne, Switzerland) was used to design, control, and monitor UAV flights. Before each flight, waypoints were predefined to achieve a minimum 70% overlap in both sideward and forward directions. All flights were conducted under stable ambient light conditions. After each flight, geo-information was acquired from the onboard GPS equipment integrated into the UAV system, and images were subsequently downloaded from an SD card for further image processing analysis.

### 2.3 Image processing

#### 2.3.1 Image mosaicking

Image mosaicking was performed using Pix4Dmapper software. The specific operation process was as follows: (1) import all images from the same date of the UAV flight into Pix4Dmapper; (2) select the coordinate system and processing options template; (3) align the raw images with altitude and spatial position information; and (4) export the orthophoto map in TIFF format. Images collected during eight other periods of maize growth were pre-processed following the afore-mentioned steps.

Additionally, the radiance reaching the lens had a linear correlation with the digital number (DN) for each band. Consequently, an empirical linear equation (Equation 1) described by Yu et al. (2016) was adopted to maintain radiometric consistency in multi-temporal images. The equation is defined as:


(1) $$DN_{\text{normalized}}=\text{a}\times DN_{\text{raw}}+\text{b}$$

where a and b are normalization coefficients derived from the reference image (July 9). These coefficients were calculated by minimizing the radiometric differences between subsequent-date images and the reference.

#### 2.3.2 VIs calculation

**Figure 2:**
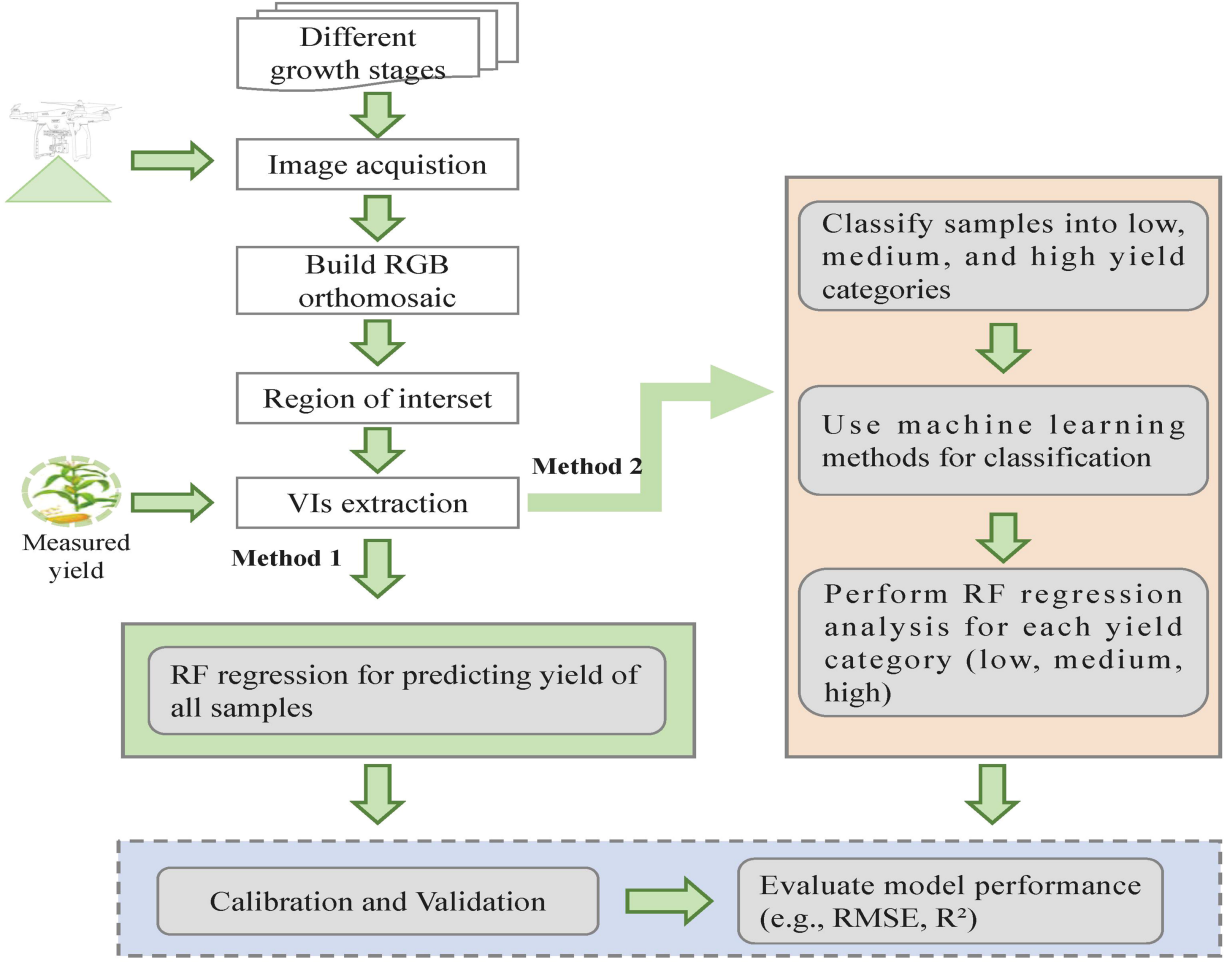
The framework for predicting maize yield in this study.

VIs were calculated from UAV-based remotely sensed orthomosaics. A total of 14 VIs, widely applied in crop research, were selected (Ge et al., 2021; Kim et al., 2018). The corresponding formulations of these VIs are provided in **Table 2**. The calculation of VIs for each plot involved three steps: (1) regions of interest (ROIs) were generated using ArcGIS v.10.8 software (ESRI, Redlands, CA, USA) to manually delineate the plots from the orthomosaics (**Figure 1**); (2) a Python script was used to calculate VIs based on the R, G, and B bands of the orthomosaics; (3) the mean VI value of each ROI was calculated using the “ZonalStatisticsAsTable” module in ArcGIS v.10.8 software.

**Table 2:** Formulations of the selected VIs in this study.

Variable	Formula	Reference
R band of UAV-based orthomosaics (R)	DN value of R band	–
G band of UAV-based orthomosaics (G)	DN value of G band	–
B band of UAV-based orthomosaics (B)	DN value of B band	–
Excess red index (ExR)	1.4R-G	(Woebbecke et al., 1995)
Excess green index (ExG)	2G-R-B	
Normalized red index (NRI)	R/(R+G+B)	(Liu et al., 2019)
Normalized green index (NGI)	G/(R+G+B)	
Normalized blue index (NBI)	B/(R+G+B)	
Green–Red ratio index (G/R)	G/R	(Maimaitijiang et al., 2019)
Green–Blue ratio index (G/B)	G/B	
Red–Blue ratio index (R/B)	R/B	
Normalized Green-Red difference index (NGRDI)	(G-R)/(G+R)	(Hunt et al., 2005)
Green minus Red index (GMR)	G-R	(Wang et al., 2013)
Color intensity index (INT)	(R + G + B)/3	(Ahmad and Reid, 1996)

### 2.4 Model building

#### 2.4.1 Classification method

In this study, classification was performed using three commonly applied methods: SVM, Decision Tree (DT), and RF. The classification method with the highest accuracy in the validation set was selected as the final method for this study. Hyperparameters for each model were tuned, as detailed in **Table 3**. The models were tested and validated using 5-fold cross-validation. The key hyperparameters for each model were selected based on their influence on the classification accuracy: (1) SVM: We tested both the ‘linear’ and ‘rbf’ kernels, selecting the optimal one based on the classification results; (2) DT: Hyperparameters such as “min_samples_leaf” and “min_samples_split” were optimized for each growth stage; (3) RF: “max_depth” and “n_estimators” were optimized for each growth stage to ensure the best model fit. The classification accuracy was evaluated based on metrics such as overall accuracy and F1-score. The best model, demonstrating the highest classification accuracy, was selected for further analysis.

**Table 3:** Detail of the user-defined parameters in the classifier models with 5-fold cross validation during the calibration.

Model	Parameter	Description	Range	Stage	Optimal value
SVM	Kernel	Specifies the kernel type to be used in the algorithm	‘linear’ and ‘rbf’	Vegetative	‘linear’
				Panicle-formation	‘linear’
				Ripening	‘rbf’
				Whole	‘linear’
	C	Regularization parameter	1-10	Vegetative	5
				Panicle-formation	1
				Ripening	1
				Whole	2
DT	min_samples_leaf	The minimum number of samples required at each leaf node	1-10, with an interval of 2	Vegetative	5
				Panicle-formation	9
				Ripening	7
				Whole	9
	min_samples_split	The minimum number of samples required to split an internal node	2-10	Vegetative	4
				Panicle-formation	2
				Ripening	8
				Whole	5
RF	max_depth	The maximum depth of each decision tree	3-15, with an interval of 3	Vegetative	12
				Panicle-formation	12
				Ripening	9
				Whole	12
	n_estimators	The number of decision trees in the forest	10-100, with an interval of 10	Vegetative	40
				Panicle-formation	30
				Ripening	50
				Whole	20

#### 2.4.2 Regression method

The RF model was used to establish regression models for yield estimation. RF is an ensemble learning algorithm that aggregates the results of individual decision trees using bagging and random feature selection. The final prediction is made by averaging the outputs of all trees in the forest (Jordan and Mitchell, 2015). RF is not sensitive to collinearity between variables, which allows it to handle complex datasets and avoid overfitting, leading to high prediction accuracy. In this study, regression models were developed using UAV-based VIs and measured yield data. The RF algorithm was implemented using the “RandomForestRegressor” function in the “scikit-learn” Python package (https://scikit-learn.org/stable/). During calibration, three hyperparameters—”max_depth”, “min_samples_split”, and “min_samples_leaf”—were tuned using a grid search method with 5-fold cross-validation.

#### 2.4.3 Calibration methods

Two different procedures were implemented to predict maize yield using UAV-based VIs (**Figure 2**). The detailed descriptions of these two strategies are as follows:

Method 1 (Regression models using the full sample set): The complete set of VIs from **Table 2** was used as input for the RF regression model. This approach aimed to predict maize yield directly from all available VI data.

Method 2 (Regression models using grouped sample sets after classification): In this method, the samples were first classified into three yield levels based on measured yield: low-level yield range (30%) corresponding to plots with the lowest yield values; medium-level yield range (40%) corresponding to plots with intermediate yield values; and high-level yield range (30%) corresponding to plots with the highest yield values. These yield levels were determined using the optimal classifier identified in Section 2.4.1. Following classification, separate RF regression models were applied to each yield group.

To optimize data collection and prediction accuracy, maize growth cycles were systematically categorized into four distinct phenological phases: vegetative stage, panicle formation stage, ripening stage, and whole growth cycle. This classification framework was specifically applied to synchronize with multi-temporal UAV observation schedules, with detailed stage-specific data acquisition timelines presented in **Table 4**.

**Table 4:** The division of different growth stages for predicting maize yield.

Specific Stage	Dates of collecting UAV-based data	Day after sowing (DAS)
Vegetative	9 July, 21 July, 27 July	68, 80, 86
Panicle-formation	5 August, 14 August, 25 August	95, 104, 115
Ripening	4 September, 18 September, 28 September	125, 139, 149
All	9 July, 21 July, 27 July, 5 August, 14 August, 25 August, 4 September, 18 September, 28 September	68, 80, 86, 95, 104, 115, 125, 139, 149

#### 2.4.4 Statistical analysis

In this study, the Pearson correlation coefficient (r) was used to analyze the relationships between UAV-based VIs and grain yield at different growth stages. The selected VIs and the yield data were used to create the raw dataset matrix with VIs as the independent variables (X) and grain yield as the dependent variable (Y). Before classification and regression, the dataset matrix was randomly split into training (70%) and testing (30%) sets for each of the four growth stages.

Additionally, two metrics, overall accuracy and F1-score, were selected to assess the accuracies of the different classifiers. Finally, the predictive performance of RF regression models was quantitatively evaluated using the coefficient of determination (R^2^) and root mean square error (RMSE). These statistical metrics were calculated in Equations 2, 3:


(2) $$R^{2}=1-\frac{\sum_{i=1}^{n}{\left(y_{i}-\hat{y_{i}}\right)}^{2}}{\sum_{i=1}^{n}{\left(y_{i}-\bar{y}\right)}^{2}}$$

(3) $$RMSE=\sqrt{\frac{\sum_{i=1}^{n}{\left(y_{i}-\hat{y_{i}}\right)}^{2}}{n}}$$

where $y_{i}$ is the measured yield, $\hat{y_{i}}$ is the predicted yield, $\bar{y_{i}}$ is the mean value of all the measured yield and n is the number of samples.

## 3 Results

### 3.1 Correlation analysis of VIs and maize yield

**Figure 3:**
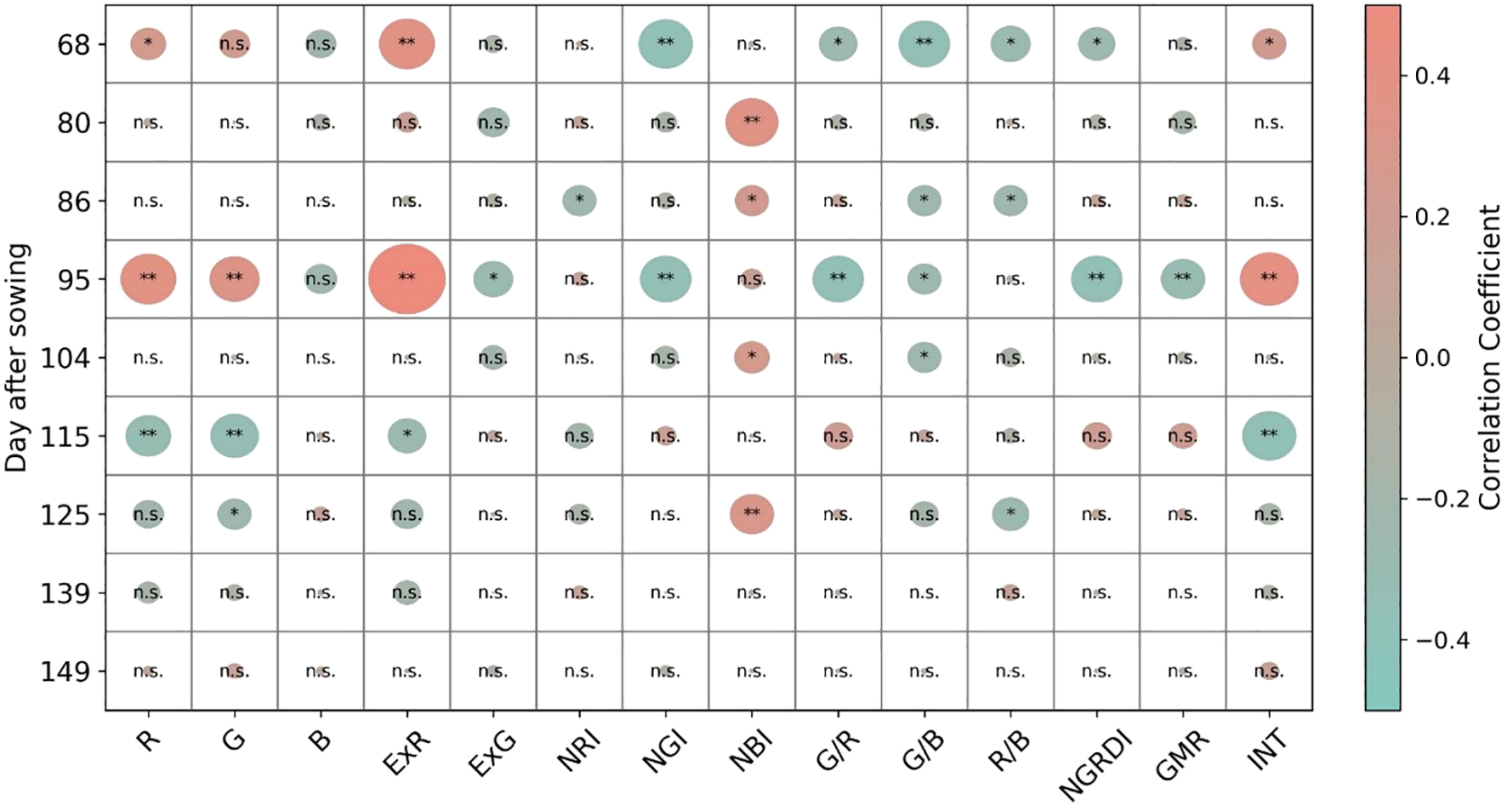
Pearson correlations between UAV-based VIs and yield in maize breeding across different growth periods. n.s., *, and ** represent ‘not significant’, p<0.05, and p<0.01, respectively.

Pearson correlation analysis was conducted to explore the relationship between maize yield and various VIs across different growth periods (**Figure 3**). The analysis revealed significant variations in the strength and significance of these correlations at different growth stages. At the early growth stages (DAS=68, 80, and 86), many VIs exhibited significant correlations with maize yield, particularly at DAS=68. Notably, ExR and NGI indices demonstrated the strongest correlations with yield at this stage, with r values of 0.39 and -0.38, respectively (**Supplementary Table 1**). This suggests that these indices are effective predictors of yield potential during the early stages of crop development. During the middle growth stages (DAS=95, 104, and 115), the correlation coefficients between VIs and yield varied more significantly across different periods. For instance, ExR exhibited the strongest correlation at DAS=95 with an r value of 0.54, but its correlation strength decreased at DAS=104 and DAS=115. Indices such as R, G, G/B, and INT showed good correlations at some individual stages but exhibited random variations overall, indicating some degree of inconsistency in their correlations with yield. In the late growth stages (DAS=125, 139, and 149), the correlations between most VIs and maize yield were generally weak and not significant. All VIs had absolute correlation coefficients (|r|) below 0.26, suggesting that these indices are less suitable for predicting yield during the post-heading stage of crop development.

### 3.2 Maize yield prediction based on method 1

**Figure 4:**
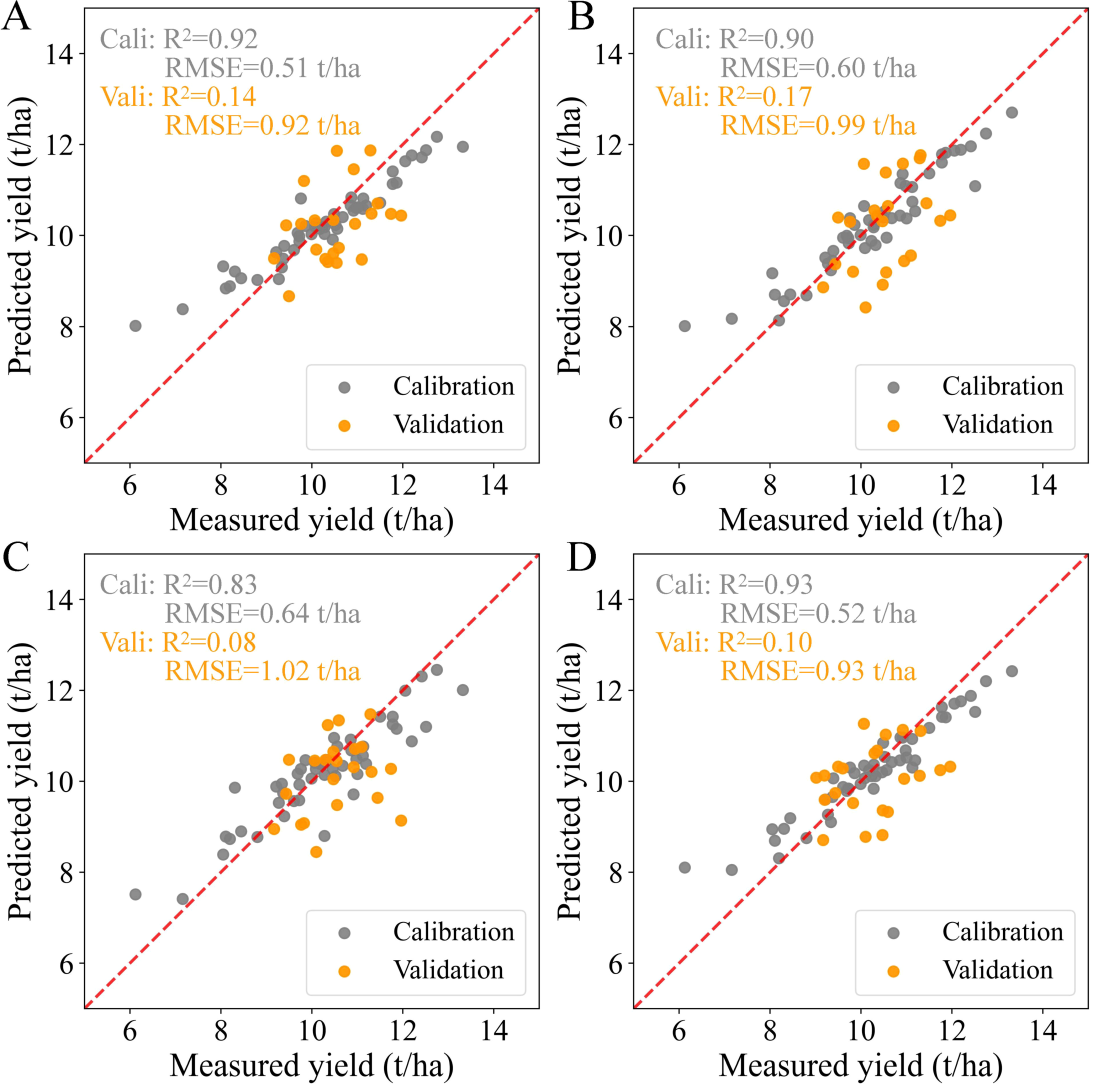
Relationship between measured and predicted yields based on Method 1 at each growth stage: **(A)** Vegetative stage, **(B)** Panicle-formation stage, **(C)** Ripening stage, and **(D)** Whole growth period. The scatterplots include results from both the calibration set and the validation set.

The results of predicted yield calculated by Method 1 at each growth stage are presented in **Figure 4**. In the calibration datasets, the RF models at the vegetative stage and combined stages achieved slightly better results than those obtained at the panicle-formation and ripening stages, with R^2^ values ranging from 0.92 to 0.93 and RMSE values ranging from 0.51 t/ha to 0.52 t/ha. The predicted yield was overestimated at different growth stages when the measured yield was less than 8.4 t/ha. In contrast, all the predicted yields were underestimated when the measured yield exceeded 10.9 t/ha. Compared to the models in the calibration set, the RF models at each growth stage showed lower accuracies and higher RMSE values in the validation set, with the scatter distributions calculated by these RF models deviating further from the 1:1 line. Although the optimal models for yield prediction were obtained at the vegetative and combined stages, the prediction accuracies were somewhat unacceptable, with R^2^ values of 0.14 and 0.10 and RMSE values of 0.92 t/ha and 0.93 t/ha, respectively.

### 3.3 Maize yield prediction based on method 2

The classification performance of three machine learning methods, including SVM, DT, and RF, was evaluated for yield-level classification across different growth stages, based on the validation set results presented in **Table 5**. SVM generally outperformed DT and RF, achieving the highest F1-score of 0.71 and an overall accuracy of 68% at the vegetative stage. At the panicle-formation stage, SVM also demonstrated better performance, with an F1-score of 0.69 and an overall accuracy of 64%, compared to DT’s F1-score of 0.60 and RF’s F1-score of 0.62. In the ripening stage, SVM maintained an F1-score of 0.56, but its overall accuracy of 50% was lower than RF’s 57%. Across the whole growth stage, SVM achieved an F1-score of 0.61 and an overall accuracy of 59%, which was higher than DT’s 57%. These results indicate that SVM is more effective for yield-level classification in maize breeding, particularly at the vegetative stage, which is crucial for early yield prediction.

**Table 5:** The accuracy of yield-level classification by using the three machine learning methods at each growth stage.

Model	Vegetative		Panicle-formation		Ripening		Whole	
	F1-score	Overall accuracy	F1-score	Overall accuracy	F1-score	Overall accuracy	F1-score	Overall accuracy
SVM	0.71	68%	0.69	64%	0.56	50%	0.61	59%
DT	0.40	43%	0.60	62%	0.45	48%	0.53	57%
RF	0.61	62%	0.62	62%	0.55	57%	0.43	43%

**Figure 5:**
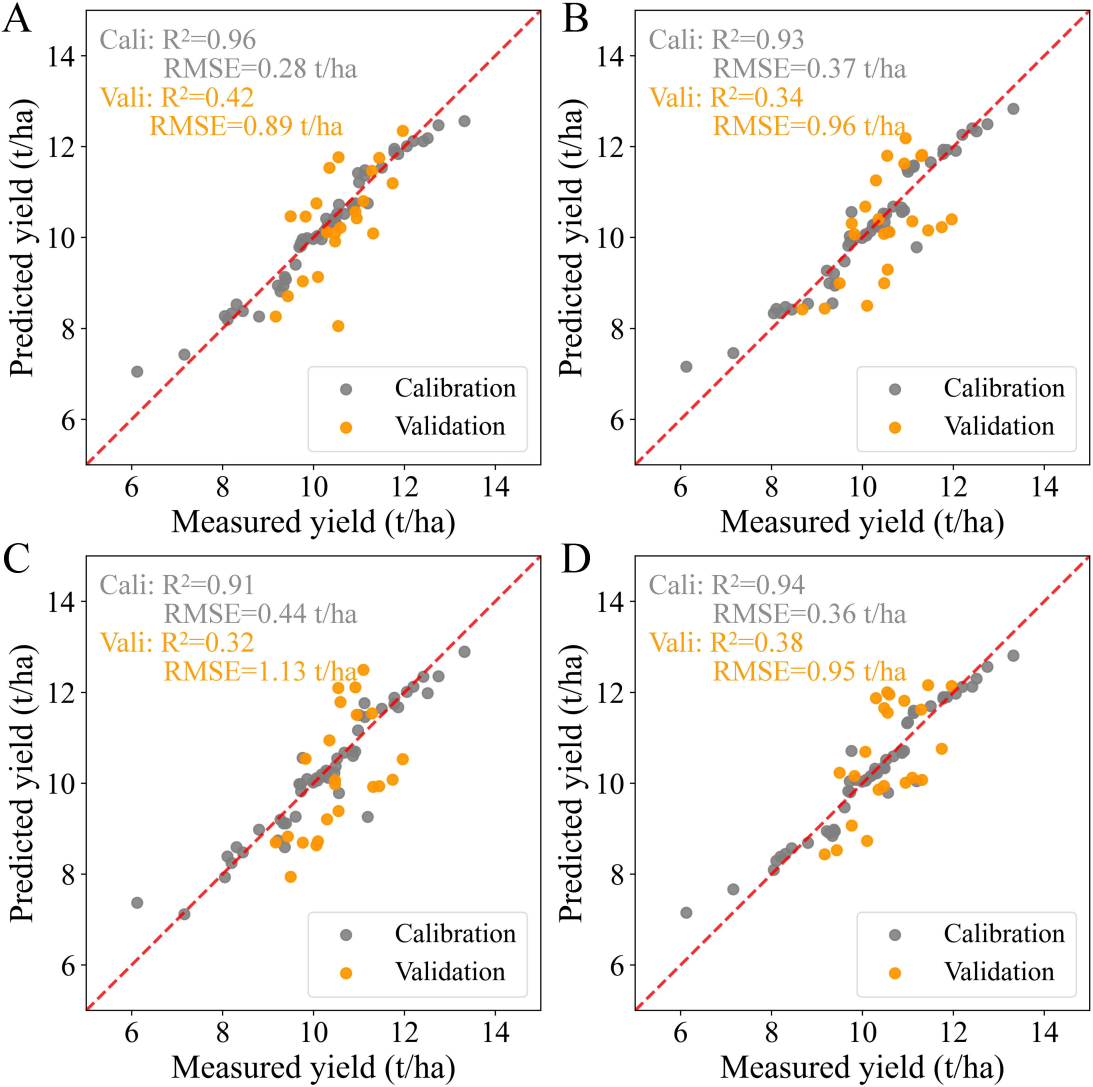
Relationship between measured and predicted yields based on Method 2 at each growth stage: **(A)** Vegetative stage, **(B)** Panicle-formation stage, **(C)** Ripening stage, and **(D)** Whole growth period. The scatterplots include results from both the calibration set and the validation set.

After selecting SVM for its superior classification performance, all yield samples were classified into three levels. For each level, the RF regression model at each growth stage was applied to predict yield. **Figure 5** shows the quantification results of Method 2. In the calibration set, there was a good relationship between measured yield and predicted yield (R^2^ = 0.91-0.96 and RMSE = 0.28-0.44 t/ha), indicating robust performance in predicting yield using Method 2 at each growth stage. Generally, the scatter distributions at each growth stage were close to the 1:1 line. In the validation set, the agreement between measured and predicted yield at the vegetative stage was better than at other stages, with R^2^ and RMSE values of 0.42 and 0.89 t/ha, respectively. This result indicated that yield simulation at the early growth stage was more reasonable due to higher prediction accuracy of yield. However, Method 2 in the validation set provided less accurate quantification results than at the same growth stage in the calibration set. Additionally, the regression models at each growth stage tended to underestimate yield when low levels of measured yield occurred.

### 3.4 Method 1 vs. Method 2

**Figure 6:**
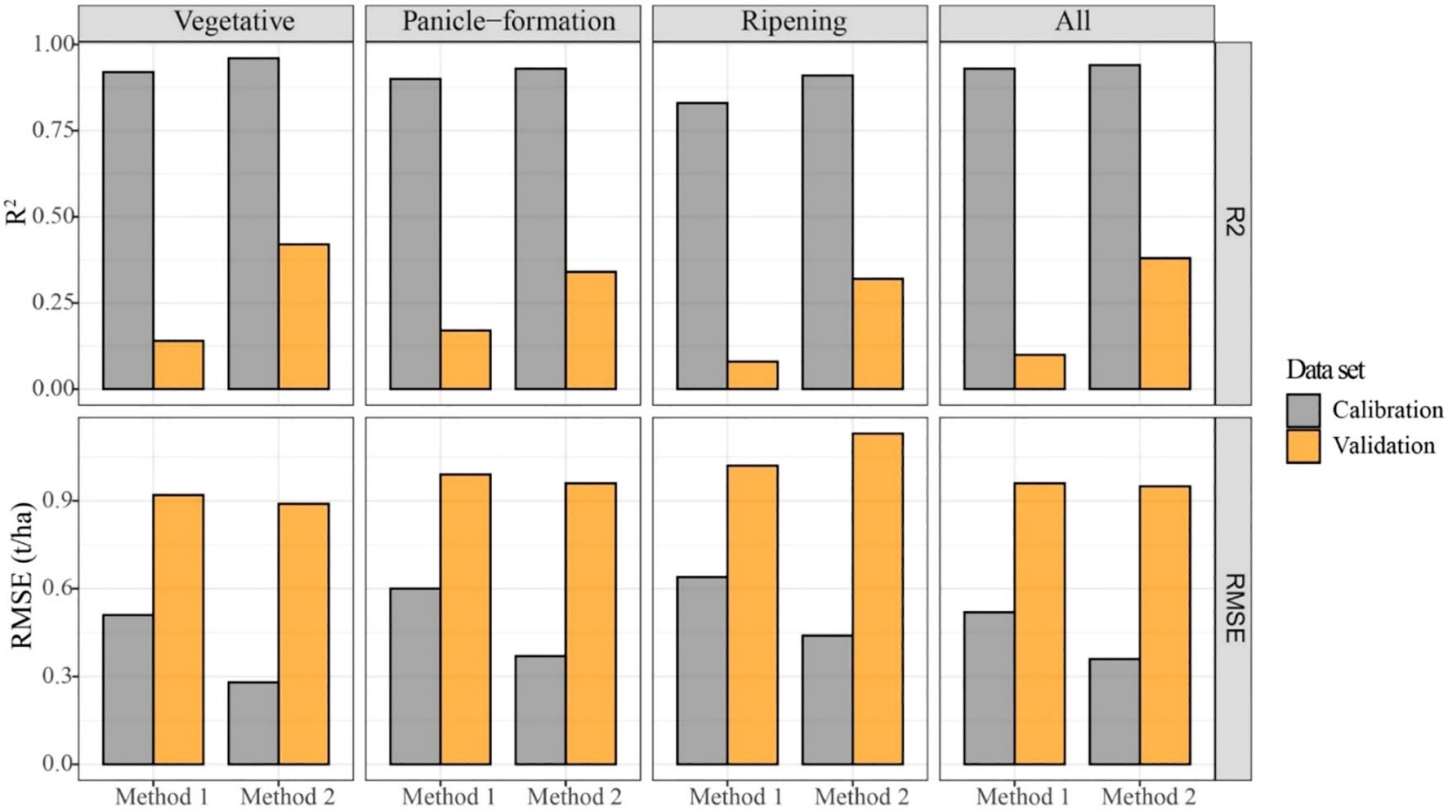
The comparison of the yield prediction accuracy by using Method 1 and Method 2 at each growth stage.

**Figure 6** illustrates the comparison of prediction results between Method 1 and Method 2 across different growth stages. As shown in the figure, Method 2 achieved significantly higher R^2^ values and lower RMSE values compared to Method 1 during the vegetative stage, indicating a marked improvement in prediction accuracy. For instance, in the calibration set, Method 2 achieved R^2^ values of 0.91-0.96 and RMSE values of 0.28-0.44 t/ha, demonstrating robust performance. In the validation set, the agreement between measured and predicted yield at the vegetative stage was better than at other stages, with R^2^ and RMSE values of 0.42 and 0.89 t/ha, respectively. These results highlight the effectiveness of the classification-before-regression strategy in enhancing yield prediction accuracies, particularly during the early growth stages.

## 4 Discussion

### 4.1 The optimal growth stage for predicting maize yield

One of the primary objectives of this study was to investigate the optimal growth stage for collecting UAV-based VIs suitable for predicting yield in maize breeding. The UAV-based VIs data were collected at the vegetative (DAS = 68, 80, and 86), panicle-formation (DAS = 95, 104, and 115), and ripening (DAS = 125, 139, and 149) stages. These growth stages are known as critical periods in maize development and growth since various stresses during these times can significantly impact yield. As shown in **Supplementary Table 1** and **Figure 3**, UAV-based VIs had strong correlations with yield during the vegetative period across different maize cultivars. For example, certain color indices such as ExR and NGI demonstrated strong correlations with yield at early growth stages (e.g., DAS=68), highlighting their potential as effective predictors of yield potential during this period.

The vegetative stage is considered the critical phase for maize development as it is during this period that the plant’s biomass accumulates rapidly, laying the foundation for subsequent growth and yield formation. During this stage, the plant is highly sensitive to environmental stresses, and any adverse conditions can significantly impact its growth and ultimately the yield. Therefore, early prediction of yield during the vegetative stage can provide valuable information for farmers to make timely decisions regarding field management practices, such as fertilization and irrigation, to optimize yield. The strong correlations between UAV-based VIs and yield during the vegetative stage suggest that this period is an optimal time window for predicting maize yield. This finding is consistent with previous studies that have shown the potential of UAV-based VIs for yield prediction during the early growth stages of crops. For instance, a study by Yang et al. (2022) found that the optimal phenological phase for maize yield prediction using high-frequency UAV remote sensing was during the vegetative stage.

In addition, the relatively weak correlation between UAV-based VIs and yield during the post-heading stage (**Figure 3**) is consistent with the findings of Zheng et al. (2019). This is mainly because the emergence of maize panicles alters canopy structure, significantly influencing the relationship between UAV-based VIs and yield. During the post-heading stage, maize genotypes consist of stems, leaves, and panicles, with both leaves and panicles contributing to canopy reflectance. Thus, VIs calculated from maize canopy reflectance exhibited varying correlations with yield. Furthermore, panicle traits (such as number, length, and weight) vary across cultivars, influencing canopy structure differently. Consequently, the correlation between VIs and yield becomes more complex for different cultivars in the post-heading stage. By contrast, as mentioned above, UAV-based VIs at the vegetative stage demonstrated better performance in correlating with yield. Therefore, it is more informative to predict yield in maize breeding during the early growth stage.

However, the relationship between single-stage VIs and yield is still affected by differences in maize cultivars. Although all maize cultivars were sown simultaneously, the maize in each plot was not at a consistent phenological stage on the imaging and field sampling days. Phenological variations can increase spatial variability. Therefore, cultivars can significantly impact maize grain yield prediction using UAV-based data. Previous studies on rice (Duan et al. 2021) and wheat (Dong et al. 2020) have investigated the influence of cultivars on crop yield prediction. The varying morphology of crop cultivars makes grain yield prediction more inaccurate and complicated. Therefore, the phenological influence of different cultivars should be considered in further analysis.

### 4.2 Improved RF regression based on SVM classification

**Figure 7:**
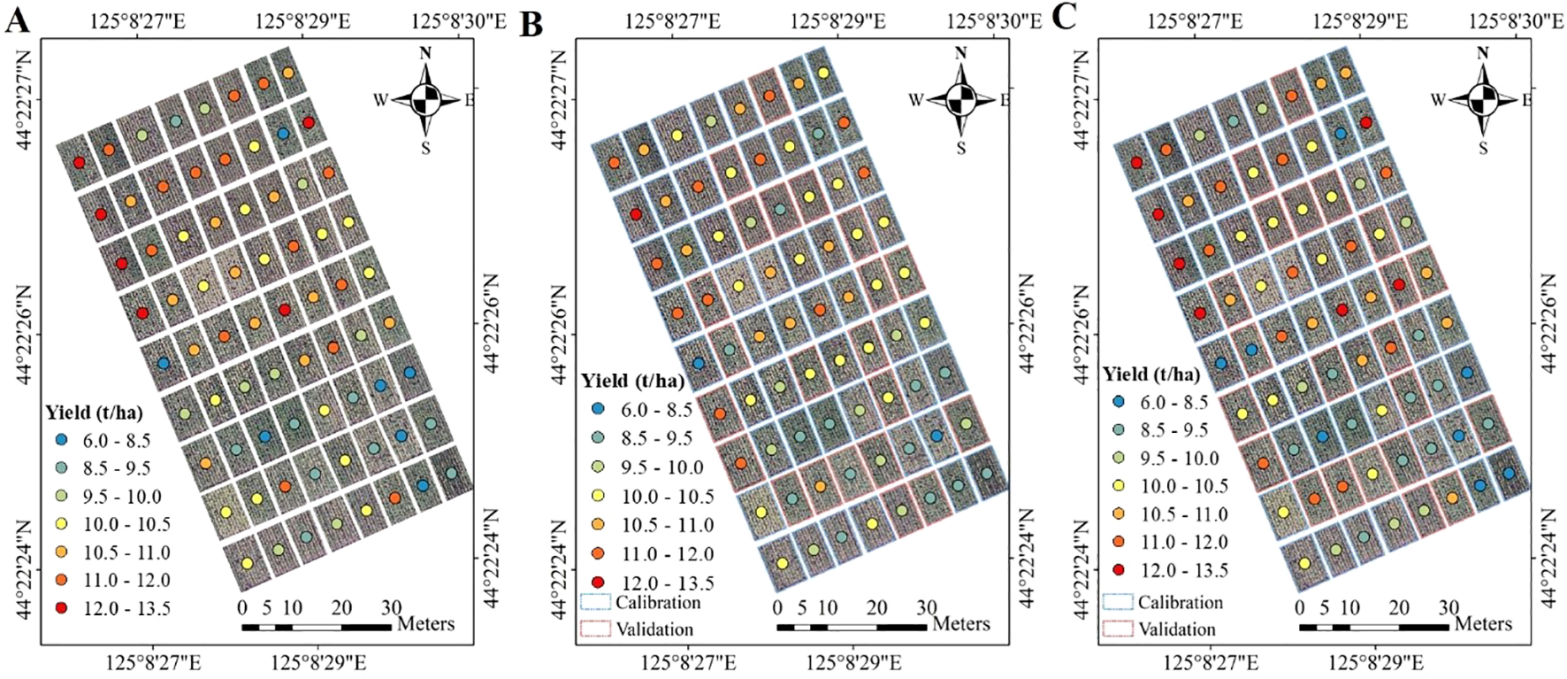
Yield maps in the maize breeding; **(A)** measured maize yield, **(B, C)** predicted yield from the Method 1 and Method 2 at the vegetative stage in the calibration and validation sets, respectively.

In this study, the performance of the two quantification methods (Method 1 and Method 2) was evaluated using R² and RMSE, with the detailed results presented in **Figure 6**. As discussed, Method 2 outperformed Method 1, particularly during the vegetative and panicle-formation stages. This improvement can be attributed to the higher classification accuracy achieved for each yield class in maize breeding during the pre-heading period (**Table 5**). The categorization into yield classes reduced the uncertainty in yield predictions by narrowing the yield range within each class, thereby enhancing the model’s performance (**Figure 7**).

Considering the large number of maize genotypes in breeding programs, which may number in the hundreds, it is crucial to explore how classification strategies impact yield prediction models. In the case of Method 2, the 72 maize yield samples were categorized into three types using the optimal classifier method (SVM), with the detailed classification results presented in **Table 5**. Among the four growth periods, the vegetative stage provided the best classification results, with overall accuracies of 68% for the validation set. Scatter plot analysis showed that the improvement in prediction accuracy with Method 2 was particularly significant for medium and high yield levels. These findings align with those of Wang et al. (2014), who demonstrated that models built on classified sample sets performed better than those using the full sample set. This suggests that SVM classification can enhance RF regression performance by improving the accuracy of models that categorize maize genotypes into distinct yield levels.

However, the lower validation accuracies compared to calibration accuracies point to several challenges. First, the use of a single year’s data resulted in a limited sample size, which may have led to overfitting during the calibration stage. Overfitting occurs when a machine learning model learns the training data too precisely, capturing noise and random fluctuations rather than the underlying patterns, which negatively affects its performance on unseen data, as observed in the validation set. Second, the significant phenotypic variation among maize cultivars resulted in different phenological stages on the imaging and field sampling days. This increased spatial variability complicated the relationship between VIs and yield, making it more difficult to characterize yield accurately using color features. Furthermore, while Method 2 shows potential for improving yield predictions in maize breeding, it may encounter limitations in large-scale applications due to the variability in environmental conditions and genetic traits. For instance, UAV-based data collection across diverse ecological regions may provide more reliable results and enhance model generalizability. Thus, expanding the sample size and incorporating data from various ecological regions will be critical for improving the robustness and accuracy of the model. Future research should focus on these aspects, incorporating larger, more diverse datasets and addressing overfitting risks through techniques such as cross-validation, the explainable artificial intelligence (XAI) technique or data augmentation (Naga Srinivasu et al., 2024).

In summary, Method 2 holds promise for enhancing yield prediction in maize breeding programs by improving classification and reducing uncertainty in predictions. However, for broader applicability, its effectiveness needs to be validated through further research with larger sample sizes, diverse environmental conditions, and comprehensive phenological data. The integration of phenological and environmental variables will be essential for improving the robustness and generalizability of the models, as also suggested by studies such as those by Guo et al. (2023) and Guo et al. (2021), which highlight the need for multidimensional data integration in predictive modeling for agriculture.

## 5 Conclusions

This study demonstrates the potential of UAV-based imagery in predicting grain yield for maize breeding. Multi-temporal UAV images were collected from a field experiment, and various VIs were calculated from these images to predict maize yield at four critical growth stages. The results show that the accuracy of yield prediction is higher when using UAV-based images from the early growth stages. A significant improvement in prediction accuracy was achieved by applying a classification-before-regression strategy, where the raw dataset was first grouped into three yield classes using the SVM method. RF regression models were then applied to predict yield for each class separately. This approach reduced prediction errors, with RMSE values of 0.28 t/ha in the calibration set and 0.89 t/ha in the validation set. The classification-before-regression strategy outperformed traditional regression models, demonstrating the potential of machine learning techniques in precision agriculture.

In summary, this work highlights the effectiveness of UAV-based imaging systems as a tool for gathering field-scale phenotypic data in maize breeding programs. By integrating RF regression models with SVM classification, this study offers a promising approach to predicting within-field yield variations. These findings contribute to the growing body of research in precision agriculture by showing that machine learning techniques can significantly improve yield prediction accuracy. For future research, the proposed methodology should be tested under varying climatic zones, to assess its robustness and generalizability. Furthermore, future studies could explore the use of UAV-based remote sensing data for other crops, as well as the time-dynamic information provided by multi-temporal VIs, which could further enhance prediction accuracy.

## Data Availability

The original contributions presented in the study are included in the article/**Supplementary Material**. Further inquiries can be directed to the corresponding author.
