# Supplementary material for: Enhancing yield prediction in maize breeding using UAV-derived RGB imagery: a novel classification-integrated regression approach
Source: Front Plant Sci. 2025 Mar 20;16:1511871. doi: 10.3389/fpls.2025.1511871 (PMC11965364; doi:10.3389/fpls.2025.1511871)
Supplement: Supplementary file 1 [file Table1.docx]

Supplementary Material

# Supplementary Table 1

Correlation analysis between yield and color indices across different growth periods.

| **Color index** | **Day after sowing** | | | | | | | | |
| --- | --- | --- | --- | --- | --- | --- | --- | --- | --- |
|  | 68 | 80 | 86 | 95 | 104 | 115 | 125 | 139 | 149 |
| R | 0.25^*^ | 0.07^n.s.^ | 0.01^n.s.^ | 0.39^**^ | -0.01^n.s.^ | -0.32^**^ | -0.22^n.s.^ | -0.17^n.s.^ | 0.08^n.s.^ |
| G | 0.22^n.s.^ | -0.03^n.s.^ | -0.03^n.s.^ | 0.35^**^ | -0.05^n.s.^ | -0.34^**^ | -0.24^*^ | -0.13^n.s.^ | 0.12^n.s.^ |
| B | -0.22^n.s.^ | -0.13^n.s.^ | 0.02^n.s.^ | -0.23^n.s.^ | 0.01^n.s.^ | 0.06^n.s.^ | 0.12^n.s.^ | -0.05^n.s.^ | 0.07^n.s.^ |
| ExR | 0.39^**^ | 0.16^n.s.^ | -0.08^n.s.^ | 0.54^**^ | -0.04^n.s.^ | -0.27^*^ | -0.23^n.s.^ | -0.19^n.s.^ | 0.04^n.s.^ |
| ExG | -0.14^n.s.^ | -0.23^n.s.^ | -0.11^n.s.^ | -0.28^*^ | -0.19^n.s.^ | 0.09^n.s.^ | -0.04^n.s.^ | -0.02^n.s.^ | -0.09^n.s.^ |
| NRI | 0.05^n.s.^ | 0.10^n.s.^ | -0.24^*^ | 0.11^n.s.^ | -0.04^n.s.^ | -0.20^n.s.^ | -0.16^n.s.^ | 0.11^n.s.^ | 0.01^n.s.^ |
| NGI | -0.38^**^ | -0.16^n.s.^ | -0.13^n.s.^ | -0.36^**^ | -0.18^n.s.^ | 0.15^n.s.^ | -0.03^n.s.^ | -0.02^n.s.^ | -0.09^n.s.^ |
| NBI | 0.05^n.s.^ | 0.37^**^ | 0.24^*^ | 0.16^n.s.^ | 0.25^*^ | 0.04^n.s.^ | 0.31^**^ | -0.05^n.s.^ | 0.04^n.s.^ |
| G/R | -0.27^*^ | -0.12^n.s.^ | 0.10^n.s.^ | -0.36^**^ | 0.07^n.s.^ | 0.21^n.s.^ | 0.08^n.s.^ | -0.05^n.s.^ | -0.04^n.s.^ |
| G/B | -0.36^**^ | -0.14^n.s.^ | -0.24^*^ | -0.24^*^ | -0.24^*^ | 0.10^n.s.^ | -0.20^n.s.^ | 0.01^n.s.^ | -0.05^n.s.^ |
| R/B | -0.28^*^ | 0.06^n.s.^ | -0.24^*^ | -0.06^n.s.^ | -0.15^n.s.^ | -0.12^n.s.^ | -0.26^*^ | 0.13^n.s.^ | -0.02^n.s.^ |
| NGRDI | -0.26^*^ | -0.12^n.s.^ | 0.10^n.s.^ | -0.36^**^ | -0.07^n.s.^ | 0.21^n.s.^ | 0.08^n.s.^ | -0.05^n.s.^ | -0.04^n.s.^ |
| GMR | -0.11^n.s.^ | -0.18^n.s.^ | 0.10^n.s.^ | -0.31^**^ | -0.09^n.s.^ | 0.20^n.s.^ | 0.09^n.s.^ | 0.01^n.s.^ | -0.06^n.s.^ |
| INT | 0.24^*^ | -0.01^n.s.^ | -0.01^n.s.^ | 0.41^**^ | -0.05^n.s.^ | -0.38^**^ | -0.17^n.s.^ | -0.12^n.s.^ | 0.14^n.s.^ |

**Note:** n.s., ^*^, and ^**^ represent ‘not significant’, p<0.05, and p<0.01, respectively.
